# Genetic regulation of glucoraphanin accumulation in Beneforté® broccoli

**DOI:** 10.1111/nph.12232

**Published:** 2013-04-08

**Authors:** Maria H Traka, Shikha Saha, Stine Huseby, Stanislav Kopriva, Peter G Walley, Guy C Barker, Jonathan Moore, Gene Mero, Frans den Bosch, Howard Constant, Leo Kelly, Hans Schepers, Sekhar Boddupalli, Richard F Mithen

**Affiliations:** 1 Food & Health Programme, Institute of Food ResearchNorwich Research Park, NR4 7UA, UK; 2 Metabolic Biology, John Innes CentreNorwich Research Park, NR4 7UH, UK; 3 Warwick Life Sciences, The University of WarwickWellesbourne, Warwick, CV35 9EF, UK; 4 Warwick Systems Biology, The University of WarwickCoventry, CV4 7AL, UK; 5 Seminis Vegetable Seeds, Inc.Arroyo Grande, CA, 93420, USA; 6 Seminis Vegetable Seeds, Inc.Wageningse Afweg 31, 6702 PD, Wageningen, the Netherlands; 7 Monsanto Center for Food and Nutrition Research, Seminis Vegetable Seeds, Inc.Kannapolis, NC, 28081, USA; 8 Seminis Vegetable Seeds, Inc.Woodland, CA, 95695, USA

**Keywords:** Beneforté® broccoli, *Brassica villosa*, glucoraphanin, glucosinolates, Myb28, S-methylcysteine sulphoxide, sulforaphane, sulphate assimilation

## Abstract

- Diets rich in broccoli (*Brassica oleracea* var *italica*) have been associated with maintenance of cardiovascular health and reduction in risk of cancer. These health benefits have been attributed to glucoraphanin that specifically accumulates in broccoli. The development of broccoli with enhanced concentrations of glucoraphanin may deliver greater health benefits.
- Three high-glucoraphanin F_1_ broccoli hybrids were developed in independent programmes through genome introgression from the wild species *Brassica villosa*. Glucoraphanin and other metabolites were quantified in experimental field trials. Global SNP analyses quantified the differential extent of *B. villosa* introgression
- The high-glucoraphanin broccoli hybrids contained 2.5–3 times the glucoraphanin content of standard hybrids due to enhanced sulphate assimilation and modifications in sulphur partitioning between sulphur-containing metabolites. All of the high-glucoraphanin hybrids possessed an introgressed *B. villosa* segment which contained a *B. villosa Myb28* allele. Myb28 expression was increased in all of the high-glucoraphanin hybrids. Two high-glucoraphanin hybrids have been commercialised as Beneforté® broccoli.
- The study illustrates the translation of research on glucosinolate genetics from *Arabidopsis* to broccoli, the use of wild *Brassica* species to develop cultivars with potential consumer benefits, and the development of cultivars with contrasting concentrations of glucoraphanin for use in blinded human intervention studies.

## Introduction

**Fig. 1:**
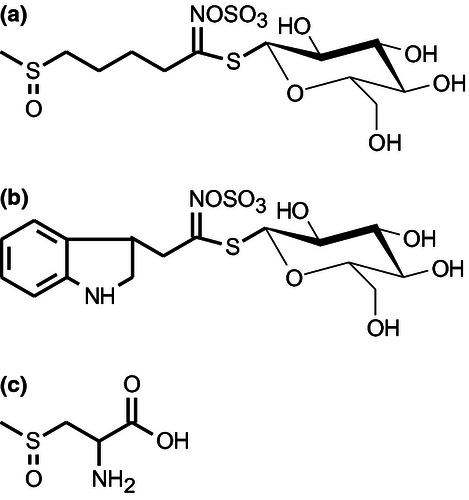
(a) 4-Methylsulphinylbutyl glucosinolate (glucoraphanin) derived from methionine and the precursor of isothiocyanate sulforaphane. (b) 3-Indolylmethyl glucosinolate derived from tryptophan. Tryptophan-derived glucosinolates do not form stable isothiocyanates upon hydrolysis. (c) S-Methylcysteine sulphoxide which is thermally degraded to several volatile sulphur-containing compounds upon cooking which are largely responsible for the ‘sulphurous’ odour of cooked *Brassica* vegetables.

Epidemiological studies have associated diets rich in cruciferous vegetables such as heading broccoli or calabrese (*Brassica oleracea* L. var *italica* Plenck) with reduced incidence of myocardial infarction (Cornelis *et al*., 2007), cardiovascular related mortality (Zhang *et al*., 2011) and reduced incidence or progression of various cancers, including lung, bowel, kidney, breast and prostate (Seow *et al*., 2002; Hsu *et al*., 2007; Kirsh *et al*., 2007; Lam *et al*., 2010; Bosetti *et al*., 2012). Significant levels of protection are most frequently observed in people that consume several portions per week, which is typical of traditional diets in parts of Asia, but is atypical of western diets (Davis *et al*., 1993; Seow *et al*., 1998). Cell and animal studies have provided evidence that degradation products of glucosinolates (sulphur-containing glycosides that specifically accumulate within these vegetables, Fig. 1) can mediate these health benefits (Juge *et al*., 2007). Despite the evidence from both epidemiological and model systems, there have been relatively few dietary intervention studies in humans to provide experimental evidence for the health-promoting activity of cruciferous vegetables, and the potential involvement of glucosinolates in mediating these effects. To facilitate these studies, we have sought to develop broccoli F_1_ hybrids that have enhanced concentrations of 4-methylsulphinylbutyl glucosinolate, commonly known as glucoraphanin. This glucosinolate is converted to the isothiocyanate sulforaphane, either due to the action of plant thioglycosidases (myrosinases) upon tissue disruption or, if cooking has denatured myrosinases, due to the action of bacterial enzymes within the gastro-intestinal tract. Sulforaphane has been shown in many cell and animal studies to have potentially health-promoting activities (Juge *et al*., 2007).

We previously described the enhanced concentration of glucoraphanin in hybrids between heading broccoli and the wild species *B. villosa* compared to either parent (Faulkner *et al*., 1998). A subsequent study reported the mapping of QTLs in segregating backcross populations derived from these F_1_ hybrids, the identification of a major QTL on linkage group 2 that determined the concentrations of methionine-derived glucosinolates, and the development of the breeding line 428-11-69 (Mithen *et al*., 2003). In this paper, we describe the use of 428-11-69 to develop three high-glucoraphanin broccoli hybrids (including two Beneforté hybrids) in three independent breeding programmes, and demonstrate the robustness of the high-glucoraphanin phenotype through an extensive series of experimental field studies.

**Fig. 2:**
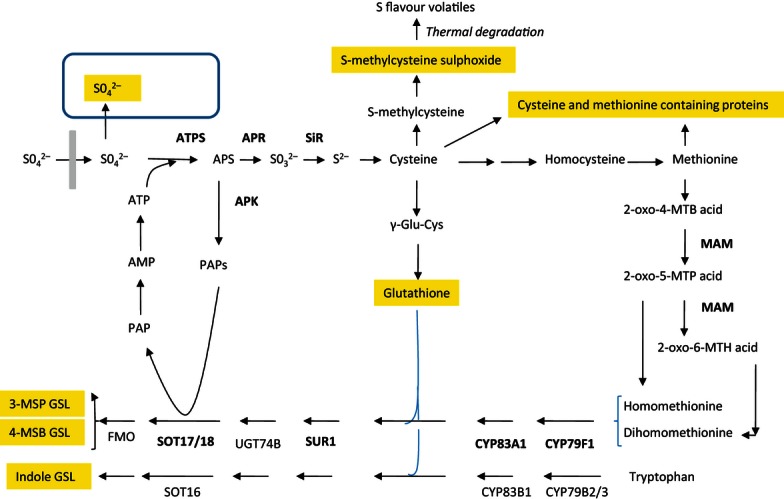
Summary of sulphur metabolism in broccoli. Sulphate, S-methyl cysteine sulphoxide, cysteine and methionine amino acids within proteins, glutathione and methionine-derived and tryptophan-derived glucosinolates are the major sulphur-containing metabolites. Sulphur pools are shown in yellow. ATPS, Adenosine triphosphate-sulphurylase; APR, APS reductase; APK, APS kinase; SiR, sulfite reductase; MAM, methylthioalkylmalate synthase; CYP, cytochrome P-450; SUR1, superroot 1; UGT, S-glycosyltransferase; SOT, Sulfotransferase; FMO, flavin-monooxygenase. Genes in bold have been shown to be upregulated by Myb28 in *Arabidopsis* (details in text)

Glucosinolates are sulphur-rich compounds, and cruciferous vegetables such as broccoli require sufficient sulphate supply to ensure yield and quality. Despite the agronomic importance of sulphur, previous studies have not defined the proportion of sulphur within glucosinolates and the other major sulphur-containing metabolites – cysteine and methionine amino acids, glutathione, sulphate (which accumulates in vacuoles) and *S*-methyl cysteine sulphoxide (SMCSO) (Figs 1, 2). The latter metabolite is thermally degraded upon cooking to produce several volatile S-containing compounds which are the major contributors to the flavour of cooked Brassica vegetables, and may cause ‘sulphurous’ off-flavours (Stoewsand, 1995). However, as with glucosinolate degradation products, SMCSO has also been associated with health-promoting activities (Komatsu *et al*., 1998; Xiao & Parkin, 2002; Helen *et al*., 2003). In addition, and also in a similar manner to glucosinolates, high concentrations of SMCSO in fodder *Brassica* can reduce palatability to livestock and cause toxicity (Stoewsand, 1995). Thus, we quantify sulphur partitioning into glucosinolates, SMCSO and other major S-containing metabolites and explore whether the high-glucoraphanin trait is due to re-partitioning of sulphur between these S pools, or is due to additional sulphur assimilation. Through comparative mapping we identify a common introgressed segment coincident with our previously described QTL that regulates glucosinolate accumulation in each of the three F_1_ hybrids. Through comparative genomics, we identified the presence of a *B. villosa* allele of the Myb28 transcription factor, an important regulator of sulphate assimilation and methionine-derived glucosinolate biosynthesis (Gigolashvili *et al*., 2007; Sonderby *et al*., 2007), that had been transferred into each of the high-glucoraphanin hybrids within this introgressed segment, and describe its expression in field grown broccoli.

## Materials and Methods

### Development of high-glucoraphanin broccoli F_1_ hybrids

**Fig. 3:**
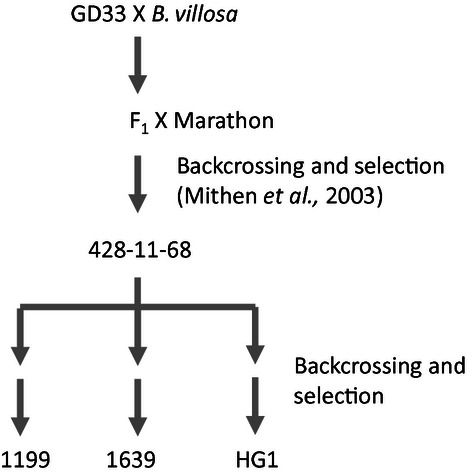
Summary of breeding programmes using the 428-11-69 line which was derived from a cross between broccoli and *Brassica villosa*, as described previously.

The broccoli (*Brassica oleracea* L. var *italic* Plenck) breeding line 428-11-69 (Mithen *et al*., 2003), derived from a cross between a double haploid broccoli breeding line and *B. villosa* Biv. (Fig. 3), was used in breeding programmes in the USA, the Netherlands and the UK. Within each programme a series of 3–5 further backcrosses and inbreeding was undertaken combined with selection for the high-glucosinolate trait, agronomic performance and quality. Breeding lines were not exchanged during the separate programmes. The high-glucoraphanin F_1_ hybrids from the US and Netherlands programme – identified as 1639 and 1199, respectively – are commercialized as Beneforté® broccoli, whereas an experimental high-glucoraphanin F_1_ hybrid from the UK programme, identified as HG1, has been used in human intervention studies (Gasper *et al*., 2005; Traka *et al*., 2008). Beneforté® is a registered trademark of Seminis Vegetable Seeds, Inc.

### Phenotypic assessment of the high-glucoraphanin trait

The high-glucoraphanin F_1_ broccoli hybrids HG1, 1199, 1639 and the standard broccoli F_1_ hybrids Emerald, Belstar, Arcadia, Fiesta and Ironman were grown under normal agronomic conditions in an experimental field plot in Norwich in 2011. Twenty plants of each variety were grown in a randomized design, and six randomly selected heads of each variety were harvested at a stage equivalent to commercial maturity, freeze dried and ground to a fine powder. Fresh florets from each plant were also retained for analyses of SMCSO. Methionine- and tryptophan-derived glucosinolates, total sulphur, sulphate, methionine, cysteine, glutathione and SMCSO were analysed in the florets of each plant, as described below. For large-scale experimental field trials, 1199 was grown along with three standard commercial broccoli varieties – Ironman, Steel and Parthenon – in 31 experimental field trials in Italy, Spain and UK in 2009, 2010 and 2011, and 1639 was grown with the two standard broccoli cultivars Heritage and Marathon in 23 experimental field trials in California, Arizona and Mexico in 2007, 2008 and 2010. Details of trial locations are provided in Supporting Information Tables S1 and S2. At each location, the broccoli was grown under standard agronomic conditions (Anon, 2010). One to three plots of each cultivar were planted, and 1–3 head samples per plot were taken for glucosinolate analyses. For each sample, three randomly selected heads of each variety were harvested at commercial maturity (125 mm–200 mm diameter head), the florets separated (taking *c*. 60% of total crown weight), pooled, freeze-dried and ground to a fine powder.

### Glucosinolate and sulphur metabolite analyses

From large-scale experimental field trials, European samples were analysed for glucosinolates at TNO (http://www.tno.nl) and North American samples were analysed at Covance (http://www.covance.com). Norwich 2011 samples were analysed at IFR. All glucosinolates were analysed by methods based on the determination of glucosinolates in Rapeseed ISO 9167-1 with some modifications as previously described (Saha *et al*., 2012). Other major sulphur-containing metabolites (methionine, cysteine, glutathione, S-methyl cysteine sulphoxide) were analysed in florets of three plants each of 1199, 1639, HG1, Emerald and Ironman grown in Norwich in 2011. Sulphate and glutathione analysis was performed as described previously (Koprivova *et al*., 2008; Scheerer *et al*., 2010). Total sulphur, free and hydrolysed cysteine, and methionine were quantified by Europhins (http://www.europhins.co.uk). SMCSO was determined as previously described with some modifications (Kubec & Dadakova, 2009). Briefly, 2 g fresh or frozen broccoli was steeped overnight in 30 ml acidified cold methanol to allow penetration of methanol into the cellular tissue. The plant material was cut in small pieces and homogenized by using a high-speed PRO 400 tissue homogenizer (Pro Scientific Inc., Oxford, CT, USA). The sample was incubated at 70°C and mixed by vortex for 10 min every 2–3 min. After centrifugation the methanolic fraction was aliquoted into a separate tube. The remaining homogenate was further extracted using 2 × 30 ml of boiling acidified methanol with 10 min incubation. The combined methanolic extracts were concentrated to 2–3 ml under reduced pressure (40°C) and adjusted to 5 ml by addition of 20 mM borate buffer (pH 9.2). The extract was stored at −20°C until derivatization. Dansyl derivatives were prepared by mixing 100 μl of the sample extract with 250 μl of Dns-Cl reagent (10 mM dansyl chloride in acetonitrile) and 0.65 ml of 20 mM borate buffer (pH 9.2). The mixture was briefly shaken, allowed to stand at room temperature for 30 min, centrifuged at 16 200 ***g*** for 10 min and analysed by HPLC-DAD/MS method by using the positive polarity mode as described below. Dansyl derivatives were analysed using a Spherisorb ODS2 (250 × 4.6 mm i.d., 5-μm particle size) column (Waters, Milford, MA, USA) connected to a model 1100 HPLC system (Agilent Technologies, Waldbronn, Germany) comprising a binary pump, degasser, cooled autosampler, column oven, diode array and mass spectrometer detectors. Samples were eluted at 0.9 ml min^−1^ with a gradient of increasing methanol using 50 mM pH 5 ammonium acetate buffer (solvent A) and methanol (solvent B). The gradient started at 30% solution B increasing over 35 min to 40%, then over 60 min to 75% B, and then was maintained for 5 min to 75% B before finally being re-equilibrated to 30% B for 5 min. Dansyl derivatives were monitored at 250 nm, full scan and selecting ion monitoring mode.

### Single nucleotide polymorphism (SNP) analyses

Total DNA was isolated from young true leaves from Ironman, 1199, 1639, HG1 and *B. villosa*, using the DNeasy Plant Maxi kit (Qiagen Inc.). The DNA was genotyped by KBioScience, Cambridge, UK, using *Brassica oleracea* specific KASPar markers. The assay principal is described online (http://www.kbioscience.co.uk/reagents/KASP_manual.pdf). In total 1577 SNPs were assayed, each marker at least 10 kb apart; 1150 SNP markers were successfully assayed across all four samples and were used for further analysis. Genotype data were analysed using the R environment (R Development Core Team, 2007). In total, we identified 673 SNPs that were polymorphic between *B. villosa* and Ironman in at least one allele. To detect the SNP markers that were indicative of a *B. villosa* introgression within the high-glucosinolate cultivars, we identified SNP markers that were homozygous or heterozygous for the *B. villosa* alleles and were different to the Ironman alleles. The SNP markers were aligned to an unpublished high-density SNP map constructed using data from the AGDH population (P. G. Walley, personal communication). The map includes the previously mapped public markers that have been mapped in the AGDH population (Sebastian *et al*., 2000). The public markers facilitated the formation of syntenic links between the previously published QTL data (Mithen *et al*., 2003) and the new SNP data. The RFLP marker pO119 on chromosome 2 is tightly linked to the QTL for concentrations of methionine-derived glucosinolates, this marker is tightly linked to a group of SNPs on chromosome 2 that delineate the *B. villosa* introgression present in 1199, 1639, HG1 but absent in Ironman.

### *Myb28* sequencing

Primers were designed against the Myb28 (Bra029311) sequence identified at the BRAD *Brassica* database (Cheng *et al*., 2011) using Primer3 v0.4.0 (Rozen & Skaletsky, 2000) and purchased from MWG UK:

*Myb28* Forward 5′-TCACGAACATGGAGAAGGTG-3′,
*Myb28* Reverse 5′-TGAGCTTGACCGGGAGTATC-3′.

DNA isolated as described above was used. Reactions were performed in 20 μl volumes containing 1X Green GoTaq® Reaction Buffer (Promega), 2.5 mM MgCl_2_, 0.2 mM dNTPs, 0.2 μM primers, 0.5 units GoTaq® DNA polymerase and 15–50 ng template DNA. Amplification was carried out using 35 cycles of 95°C for 30 s, 53°C for 1 min and 72°C for 1 min with a final extension at 72°C for 5 min. Ampilicons were gel purified using the QIAquick Gel Extraction Kit (Qiagen) and sequenced (TGAC, Norwich, UK).

### Real-Time RT-PCR of Myb28 and actin

The Myb28 sequence was identified as described above. A brassica actin sequence was identified using the AT3G18780.2 CDS sequence of *Arabidopsis thaliana* to BLAST the BRAD *Brassica* database (Cheng *et al*., 2011). Both assays were designed using ABI PRISM Primer Express v2 (Applied Biosystems). Primers and TaqMan probes with 5′-FAM and 3′-TAMRA modifications were purchased from MWG UK:

Myb28 For 5′-CTCTTCCTCTTTCCTCGGGTTT-3′,
Myb28 Rev 5′- TGCAACTCAAGGAACCTCTCTGA-3′,
Myb28 probe 5′-AACCCGGTTTCCGAGATCACCACAC-3′;
Actin For 5′- GCAGACCGTATGAGCAAAGAGA-3′,
Actin Rev 5′- GGGAGGTGCAACGACCTTAA3′,
Actin probe 5′- CACAGCACTTGCACCAAGCAGCATG-3′.

Total RNA from all the broccoli cultivars was extracted using a phenol-chloroform-isoamyl alcohol mixture (25 : 24 : 1) and a LiCl precipitation. cDNA was synthesized from 1 μg total RNA with QuantiTect Reverse Transcription Kit (Qiagen), which includes a DNAse step to remove possible DNA contamination. Expression of Myb28 and actin mRNA levels was determined by real time RT-PCR using the ABI Prism Step One Plus Sequence Detection System (Applied Biosystems). The real time RT-PCR reactions were carried out in 20 μl volumes using microamp optical 96-well plates. The reactions contained Taqman® RNA-TO-CT 1-Step master mix reagent kit (Applied Biosystems), 20 ng total RNA, 0.25 U μl^−1^ Multiscribe™ and optimized concentrations of primers and probes. RT-PCR conditions used were: one cycle of 48°C for 30 min, one cycle of 95°C for 10 min followed by 40 cycles at 95°C for 15 s and one cycle at 60°C for 1 min. The data for Myb28 were analysed using a standard curve generated by a serial dilution of total RNA from one Ironman plant. Actin was used as an invariant endogenous control to verify equal RNA loading.

### Nutrient analysis

Protein, total dietary fibre, β-carotene, folic acid and Vitamin C were analysed in florets of 1199 and 1639 from experimental field trials in Europe and North America by Eurofins (http://www.eurofins.co.uk) and Covance (http://www.covance.com), respectively, using standard AOAC methods (http://www.aoac.org). Additionally, Vitamin E was analysed in florets of 1639 and calcium, iron, sodium and total sugar content was analysed in florets of 1199.

### Statistical analysis

Concentrations of sulphur-containing and other metabolites, and *Myb28* gene expression were compared between cultivars by ANOVA.

## Results

### Glucosinolate expression

**Fig. 4:**
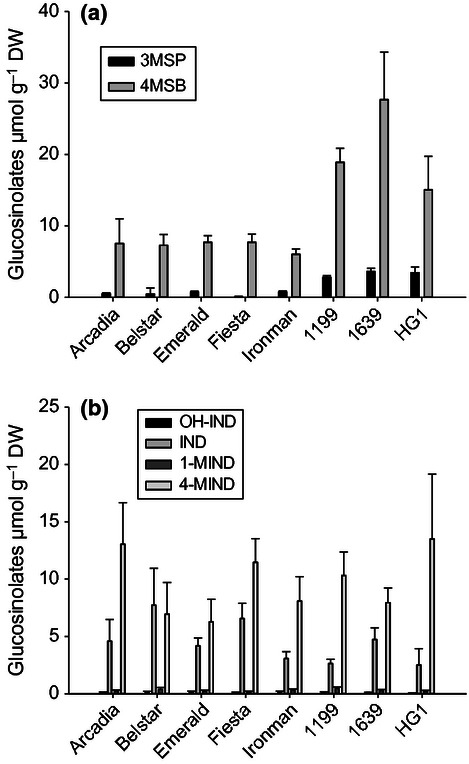
(a) Content (mean ± SD) of the methionine-derived glucosinolates, 3-methylsulphinylpropyl (3MSP) glucosinolate (glucoiberin) and 4-methylsulphinylbutyl (4MSP) glucosinolate (glucoraphanin), in florets of broccoli (*Brassica oleracea* var *italica*) F_1_ hybrids grown in Norwich in 2011. Concentrations of 3MSP and 4MSB are significantly higher in HG1, 1639 and 1199 compared to the other broccoli cultivars (*P* < 0.001). (b) Content (mean ± SD) of tryptophan-derived glucosinolates, OH-indole (OHIND), indole (IND), 1-methoxyindole glucosinolate (1-MIND) and 4-methoxyindole (4-MIND) glucosinolate

**Fig. 5:**
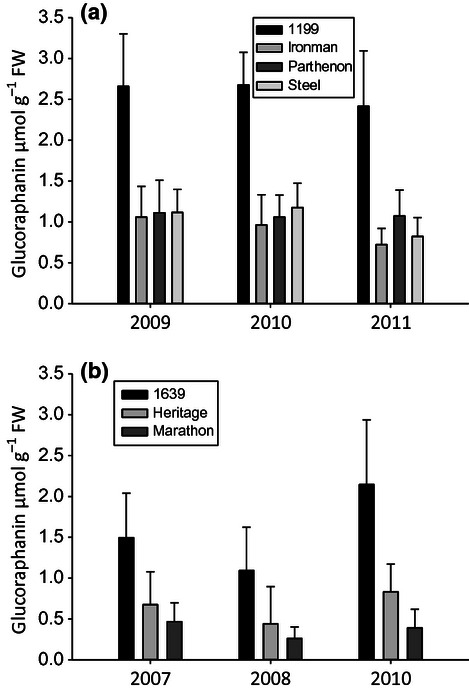
(a) Summary (mean ± SD) of glucoraphanin content of florets of broccoli 1199, Ironman, Parthenon and Steel in 31 experimental field trials undertaken in Spain, Italy and UK. The content of 1199 is significantly higher than other cultivars (*P* < 0.001). (b) Summary of glucoraphanin content (mean ± SD) of florets of 1639, Heritage and Marathon in 23 experimental field trials undertaken in California, Arizona and Mexico. The content of 1639 is significantly higher than other cultivars (*P* < 0.001).

The methionine-derived glucosinolates, glucoraphanin (4-methylsulphinylbutyl glucosinolate) and glucoiberin (3-methylsulphinylpropyl glucosinolate) were significantly higher in florets of HG1, 1199 and 1639 than standard broccoli cultivars (Fig. 4a, Table 1). There were no significant differences in tryptophan-derived glucosinolates (Fig. 4b, Table 1). Glucoraphanin was consistently higher in 1199 compared to standard broccoli cultivars in 31 experimental field trials conducted in UK, Spain and Italy (Fig. 5a), and also in 1639 compared to standard broccoli cultivars in 23 experimental field trials undertaken in California, Arizona and Mexico (Fig. 5b). High-glucoraphanin broccoli harvested from these trials had equivalent yield and floret quality to standard cultivars.

**Table 1:** Content of the major sulphur-containing metabolites in florets of five broccoli F_1_ hybrids

	Total sulphur	Methionine	Cysteine	S-methyl cysteine sulphoxide	Sulphate	Glutathione	Tryptophan-derived glucosinolates	Methionine-derived glucosinolates
Ironman	245 ± 4.9^a^	33.4 ± 1.06^a^	28.9 ± 1.84^a^	53.0 ± 3.30^a^	89.8 ± 5.27^a^	6.9 ± 0.79^a^	10.7 ± 2.95^a^	6.3 ± 0.55^a^
Emerald	282 ± 28.1^a^	33.5 ± 1.36^a^	28.7 ± 0.96^a^	53.8 ± 5.26^a^	102.2 ± 8.70^a^	6.9 ± 0.53^a^	11.4 ± 1.88^a^	8.8 ± 0.91^a^
HG1	345 ± 14.1^b^	38.7 ± 0.54^b^	30.5 ± 1.83^a^	38.2 ± 2.00^b^	98.4 ± 19.9^a^	6.9 ± 0.41^a^	11.4 ± 0.36^a^	22.9 ± 1.05^b^
1199	332 ± 20.4^b^	36.9 ± 2.35^a,b^	30.6 ± 1.69^a^	44.5 ± 1.56^b^	100.7 ± 20.6^a^	6.9 ± 0.71^a^	13.4 ± 2.17^a^	21.7 ± 1.31^b^
1639	394 ± 17.7^c^	39.4 ± 2.89^b^	32.3 ± 2.48^a^	53.7 ± 1.96^a^	112.5 ± 13.10^a^	5.6 ± 0.82^a^	13.7 ± 1.83^a^	30.1 ± 5.58^c^

Data are expressed in μmol g^−1^ DW as mean ± SD of six plants. Within columns data followed by the same letter are not significantly different (*P* < 0.05).

### Sulphur assimilation and partitioning

**Fig. 6:**
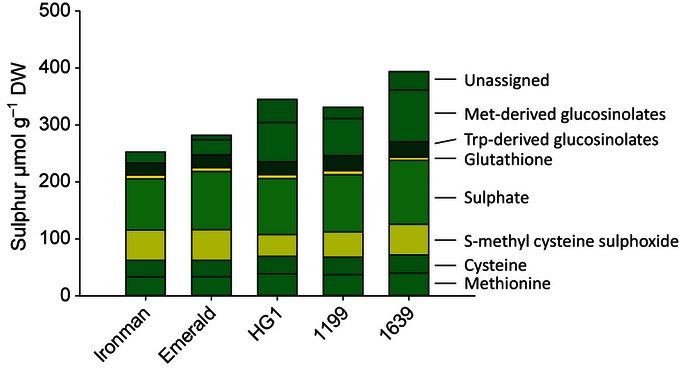
Sulphur content and sulphur partitioning between major sulphur-containing metabolites in broccoli Ironman, Emerald, 1199, HG1 and 1639. Details are provided in Tables 1 and 2.

The hybrids 1199, 1639 and HG1 had significantly higher amounts of total sulphur compared to Ironman and Emerald. 1639 also had significantly higher concentrations compared to HG1 and 1199 (Table 1, Fig. 6). Concentrations of other major S-containing metabolites are summarized in Table 1. There is a higher absolute amount of methionine in HG1 and 1639, and a lower absolute amount of *S*-methyl cysteine sulphoxide in HG1 and 1199. As methionine- and tryptophan-derived glucosinolates have three and two sulphur atoms per molecule, respectively (Fig. 1), a greater insight into sulphur partitioning comes from considering the relative proportion of total sulphur within each of the major sulphur-containing metabolites (Table 2 and Fig. 6). Thus, the enhanced concentrations of methionine-derived glucosinolates in HG1, 1199 and 1639 arises through, first, an increase in the total sulphur, through enhanced sulphate assimilation and subsequent reduction, and, second, an increase in the percentage of sulphur allocated to methionine-derived glucosinolates, and a decrease in that allocated to SMCSO (Table 2). In 1199 and HG1 this results in an absolute reduction in SMCSO, whereas in 1639 there is no reduction in absolute amount due to the greater total amount of sulphur in this hybrid.

**Table 2:** Percentage of sulphur within the major sulphur-containing metabolites in florets of five broccoli F_1_ hybrids

	% Total sulphur							
	
	Methionine	Cysteine	S-methyl cysteine sulphoxide	Sulphate	Glutathione	Tryptophan-derived glucosinolates	Methionine-derived glucosinolates	Unaccounted
Ironman	13.6 ± 0.32^a^	11.8 ± 0.78^a^	19.2 ± 2.53^a^	36.5 ± 1.43^a^	2.8 ± 0.27^a^	8.7 ± 2.40^a^	7.7 ± 0.75^a^	−2.9 ± 2.73^a^
Emerald	11.9 ± 0.94^b^	10.2 ± 0.47^a,b^	21.6 ± 1.77^a^	36.3 ± 0.76^a,b^	2.5 ± 0.30^a,b^	8.1 ± 0.69^a^	9.5 ± 1.12^a^	2.3 ± 6.86^a,b^
HG1	11.2 ± 0.32^b^	8.8 ± 0.40^b,c^	11.1 ± 1.03^b^	28.4 ± 1.44^b^	2.0 ± 0.12^b^	6.6 ± 0.07^a^	19.9 ± 1.73^b^	11.9 ± 1.22^b^
1199	11.2 ± 0.73^b^	9.2 ± 0.46^b,c^	13.5 ± 1.20^b^	30.2 ± 4.51^a,b^	2.1 ± 0.18^b^	8.1 ± 1.04^a^	19.7 ± 1.28^b^	6.1 ± 2.33^a,b^
1639	10.0 ± 0.73^b^	8.2 ± 0.49^c^	13.6 ± 0.91^b^	28.5 ± 2.80^b^	1.4 ± 0.16^c^	7.0 ± 0.82^a^	23.0 ± 4.71^b^	8.2 ± 4.90^a,b^

Data are expressed as mean ± SD. Within columns data followed by the same letter are not significantly different (*P* < 0.05).

### Nutrient analyses

In 10 experimental field trials in California, Arizona and Mexico, no differences among the key nutrients (protein, total dietary fibre, beta carotene, folic acid, Vitamin E and Vitamin C) were observed for 1639 relative to current common broccoli varieties marketed in the U.S. (Table 3). A similar result was obtained for 1199 in Europe (Table 4), with the exception of Vitamin C which was higher in Steel.

**Table 3:** Nutrient analysis of the Beneforté 1639 hybrid compared to commercial cultivars

	1639	Heritage	Ironman	Marathon
Protein (g 100 g^−1^)	3.9 ± 0.49	3.92 ± 0.51	–	4.23 ± 0.44
Total fibre (g 100 g^−1^)	3.25 ± 0.2	3.17 ± 0.23	3.3 ± 0.17	3.17 ± 0.22
β-carotene (mg 100 g^−1^)	0.79 ± 0.28	0.87 ± 0.31	0.66 ± 0.1	0.62 ± 0.15
Total folate (μg g^−1^)	1.28 ± 0.14	1.35 ± 0.16	1.45 ± 0.05	1.28 ± 0.12
Vitamin C (mg g^−1^)	0.94 ± 0.09	0.96 ± 0.09	0.97 ± 0.02	0.99 ± 0.08
Vitamin E (mg 100 g^−1^)	1.04 ± 0.25	1.03 ± 0.23	0.68 ± 0.19	1.04 ± 0.2

Data are expressed as mean ± SD in FW tissue. No significant differences were found between cultivars (*P* < 0.05).

**Table 4:** Nutrient analysis of the Beneforté 1199 hybrid compared to commercial cultivars

	1199	Ironman	Parthenon	Steel
Protein (g 100 g^−1^)	4.6 ± 1.6	4.1 ± 0.96	4.4 ± 1.64	4.4 ± 1.44
Total fibre (g 100 g^−1^)	4.4 ± 1.49	3.9 ± 0.83	4.7 ± 1.74	4.6 ± 1.71
β-carotene (mg 100 g^−1^)	0.1 ± 0.05	0.2 ± 0.07	0.1 ± 0.05	0.1 ± 0.06
Total folate (μg g^−1^)	1.0 ± 0.26^a,b,c^	1.08 ± 0.24^b,c^	0.8 ± 0.24^a^	1.1 ± 0.14^b,c^
Vitamin C (mg g^−1^)	1.2 ± 0.16^a^	1.2 ± 0.17^a^	1.2 ± 0.17^a^	1.4 ± 0.22^b^
Calcium (Ca) (mg 100 g^−1^)	71.5 ± 47.43	57.6 ± 24.38	67.5 ± 40.71	72.8 ± 50.13
Iron (Fe) (mg 100 g^−1^)	0.7 ± 0.14	0.7 ± 0.1	0.7 ± 0.21	0.7 ± 0.17
Sodium (Na) (mg 100 g^−1^)	5.0 ± 3.26	4.2 ± 1.82	5.6 ± 3.79	5.1 ± 3.4
Total sugars (g 100 g^−1^)	1.7 ± 0.67	2.1 ± 0.36	1.7 ± 0.72	2.0 ± 0.92

Data are expressed as mean (± SD) in fresh weight tissue. No significant differences were found between cultivars except for Vitamin C and total folate, where within rows data followed by the same letter are not significantly different (*P* < 0.05).

### Quantifying the extent of *B. villosa* introgression – SNP mapping

**Fig. 7:**
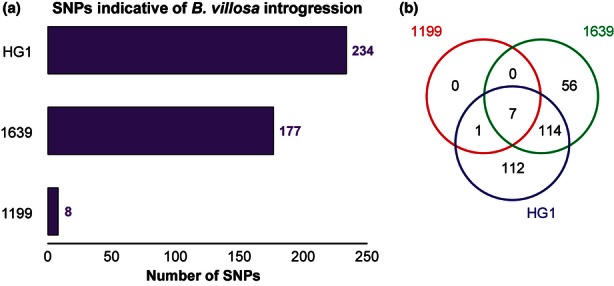
SNP analysis in the 1199, 1639 and HG1 broccoli F_1_ hybrids. (a) Number of SNPs indicative of introgression from *Brassica villosa*. (b) Overlap of introgressed SNPs in the three broccoli F_1_ hybrids.

**Fig. 8:**
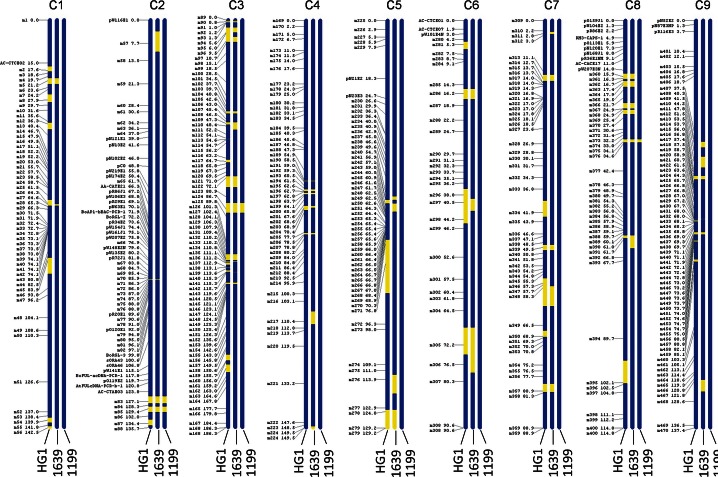
Linkage map illustrating the locations of SNPs that fall within introgressed segments (shown in yellow) of *Brassica villosa* in the HG1, 1639 and 1199 broccoli F_1_ hybrids and the broccoli background in blue. Unpublished SNP markers are prefixed ‘m’. RFLP probes are prefixed pN, pO, pR (see Mithen *et al.,* 2003), and pW; AFLP markers are labelled using the primer pairs (see Sebastian *et al.,* 2000). AtFULcDNA, BoAP1-bBAC, BoFUL-acDNA, and pCO are unpublished RFLP markers; RM-3 is an unpublished CAPS marker and sORA43 and 46 are unpublished SSR markers. BoRGL-I is a CAPS marker (Vicente & King, 2001).

In order to quantify the extent of the distribution of *Brassica villosa* genome introgression into the genomes of 1199, 1639 and HG1 we undertook global SNP genotyping using *B. oleracea* specific KASPar assays. 673 SNPS were identified that were polymorphic between Ironman and *B. villosa*. Of these, 234 *B. villosa* SNP alleles were present in HG1, 177 in 1639 and eight in 1199 (Figs 7a, 8), indicative of the different extents of introgression of the *B. villosa* genome in the high-glucoraphanin hybrids.

We sought to identify whether there were common *B. villosa* SNP alleles amongst the three high-glucoraphanin hybrids indicative of a *B. villosa* introgression that could contain alleles required for enhancing glucoraphanin accumulation. We identified seven such SNP markers (Fig. 7b) which mapped to three linkage groups. Markers mapping to linkage group 2 were genetically linked to an allele of the RFLP marker pO119 that had previously been shown to be associated with the major QTL determining methionine-derived glucosinolate accumulation in segregation populations derived from *B. villosa* (Mithen *et al*., 2003). Comparative genomic analyses with *B. rapa* suggested that this region of the genome contained the transcription factor Myb28 (Wang *et al*., 2011), which has previously been associated with determining methionine-derived glucosinolate concentrations in *Arabidopsis thaliana* (Sonderby *et al*., 2007) and *B. napus* (Harper *et al*., 2012).

### Sequencing *Myb28*

**Fig. 9:**
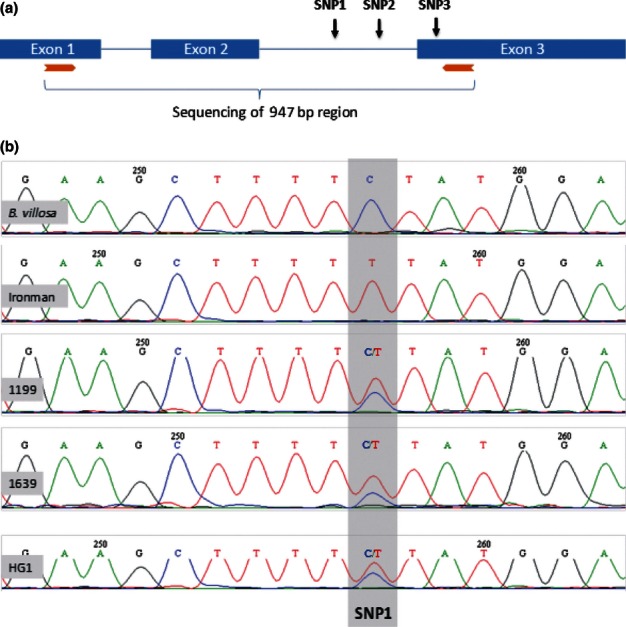
Sequencing the Myb28 transcription factor. (a) Structure of the *Myb28* gene showing the 947-bp region that was sequenced and the location of the identified SNPs. (b) Sequence of the intronic SNP1 from *Brassica villosa*, Ironman, 1199, 1639 and HG1 broccoli cultivars showing the introgression of a *B. villosa* allele in the three broccoli F_1_ hybrids, indicated by the presence of cytosine in 1199, 1639 and HG1 (shaded).

A 947-bp genomic region of *Myb28* that contained parts of all three exons constituting the *Myb28* gene (Fig. 9) was amplified from *B. villosa* (the original donor of the high-glucoraphanin trait), Ironman, HG1, 1199 and 1639 and sequenced. Three SNPs were identified that were indicative of the presence of a *B. villosa Myb28* allele in the three high-glucoraphanin hybrids; two located in the intronic region upstream of exon 3 and one at the 5′-end of exon 3 (Fig. 9).

### Expression of the Myb28 transcription factor in broccoli cultivars

**Fig. 10:**
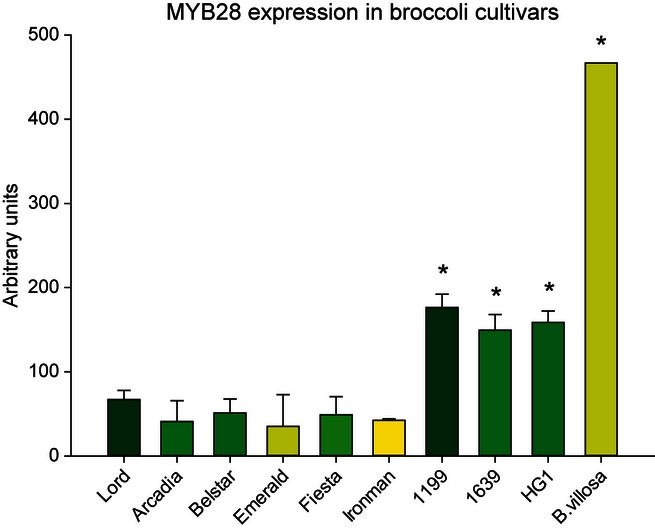
Gene expression of Myb28 in leaves of the standard broccoli cultivars, Lord, Arcadia, Belstar, Emerald, Fiesta, Ironman, the three broccoli F_1_ hybrids, 1199, 1639 and HG1, and *Brassica villosa*. Data are expressed as means ± SD (*n* = 5 for all cultivars except Lord (*n* = 3) and *B. villosa* (*n* = 1)). An asterisk indicates significantly increased expression (*P* < 0.001) relative to Ironman.

In order to determine whether expression of Myb28 is altered in different broccoli cultivars we measured its level of expression in the leaves and florets of high-glucoraphanin broccoli hybrids and standard broccoli cultivars grown in field plots in Norwich in 2011, and also in the leaves of glasshouse grown *B. villosa* and the cultivar Lord. The expression of Myb28 in leaves of the high-glucoraphanin hybrids was intermediate between that observed in the standard cultivars and *B. villosa* (Fig. 10), consistent with these hybrids being heterozygous for a ‘standard broccoli’ *Myb28* allele and a *B. villosa Myb28* allele. Expression of Myb28 in florets was more variable, with high concentrations observed in HG1, but with concentrations comparable to Ironman in 1199 and 1639 (data not shown).

## Discussion

Epidemiological studies that correlate diets rich in broccoli with health benefits, and experimental research that associates these health benefits with sulforaphane derived from glucoraphanin, suggest that broccoli cultivars with enhanced concentrations of glucoraphanin may have improved nutritional qualities. Faulkner *et al*. (1998) reported that a hybrid between broccoli and *Brassica villosa* accumulated remarkably high concentrations of glucoraphanin in floret, and that aqueous extracts of these florets in which the glucoraphanin was hydrolysed to sulforaphane were potent inducers of quinone reductase in mammalian cell assays. Subsequent breeding programmes led to the development of commercial broccoli F_1_ hybrids with enhanced glucoraphanin expression in florets derived from this original cross (Fig. 3). An extensive series of field studies demonstrated the robustness of the high-glucoraphanin trait, with a consistent 2.5–3-fold enhancement of glucoraphanin compared to standard broccoli cultivars (Figs 4, 5, Table 1), when grown under similar environmental conditions.

The enhanced concentrations of glucoraphanin could be derived by repartitioning of existing sulphur stores within broccoli, enhanced assimilation of sulphate from the soil, or redistribution of existing glucosinolates to florets from other tissues. We show that the high-glucoraphanin trait is due to two changes in sulphur metabolism. First, the high-glucoraphanin hybrids have significantly higher concentrations of total sulphur (Table 1, Fig. 6) due to enhanced sulphate assimilation, and, secondly, a higher percentage (≈ 20%) of the assimilated sulphur is channelled to methionine-derived glucosinolates than that in standard broccoli cultivars (≈ 8%) (Table 2). This increase in sulphur being channelled to methionine-derived glucosinolates was associated with a decrease of that being channelled to SMCSO (≈ 13% as opposed to 20%), resulting in a significant decrease in SMCSO in HG1 and 1199 compared to standard broccoli cultivars, but not in 1639 due to the higher concentration of total sulphur in this cultivar.

In order to elucidate the genetic basis of the high-glucoraphanin trait, we identified 673 SNPs between *B. villosa*, the donor of the high-glucoraphanin trait, and the cultivar Ironman. Of these we found 234, 177, and eight *B. villosa* SNPs in HG1, 1639 and 1199, respectively, indicative of different extents of *B. villosa* introgression in the three high-glucoraphanin hybrids. Seven of these *B. villosa* SNPs were common to each of the three F_1_ hybrids, and occurred in three clusters on linkage groups 2, 3 and 8 (Fig. 8). The cluster on linkage group 2 had previously been shown to be genetically linked to the RFLP maker pO119 which itself has been associated with the major QTL determining methionine-derived glucosinolate accumulation in broccoli (Mithen *et al*., 2003). Comparative genomics with *B. rapa* suggested the presence of the transcription factor Myb28 in the region of linkage group 2 associated with the introgressed *B. villosa* SNP alleles. Moreover, the ‘low glucosinolate trait’ in *B. napus* ssp *oleifera* (oilseed rape) that is associated with a QTL on linkage group C2 (homologous to *B. oleracea* linkage group 2) has also been associated with Myb28 (Harper *et al*., 2012). Thus, we amplified and sequenced Myb28 in *B. villosa* and the different high-glucoraphanin hybrids and showed that the high-glucoraphanin hybrids possessed one *Myb28* allele that was derived from *B. villosa*, and one allele that was derived from standard broccoli, as would be expected in an F_1_ hybrid. Furthermore, we showed that the high-glucoraphanin hybrids had consistently higher constitutive expression of Myb28 in leaves compared to the standard broccoli cultivars. This is consistent with leaves being the major source of methionine-derived glucosinolates that are transported into the florets, as has been shown in *Arabidopsis* (Chen *et al*., 2001; Brown *et al*., 2003). In contrast to expression in leaves, the expression of Myb28 in florets was more variable between the high-glucoraphanin hybrids. Studies in *Arabidopsis* have suggested that reproductive tissues are not major sites of glucosinolate biosynthesis (Redovnikovic *et al*., 2012).

The Myb28 transcription factor mapped in *B. rapa* is genetically linked to two putative methylthioalkylmalate (*MAM*) synthase genes that may be involved in the biosynthesis of methionine-elongated homologues as precursors of glucosinolates (Fig. 2) (Wang *et al*., 2011). It is conceivable that a novel *B.villosa MAM* allele may have been introgressed along with the *B. villosa Myb28* allele into the high-glucoraphanin broccoli. A novel *MAM* allele is, however, unlikely to be the cause of the high-glucoraphanin trait as there is no evidence that modification of *MAM* gene expression can affect sulphur uptake and metabolism, or lead to enhanced concentrations of methionine-derived glucosinolates as opposed to changing the ratio of side chain lengths (Field *et al*., 2004; Textor *et al*., 2007).

Thus, both genetic and gene expression studies indicate that the high-glucoraphanin trait is likely to be due to the introgression of a *B. villosa Myb28* allele into a standard broccoli genetic background. In particular, the remarkably small extent of the *B. villosa* genome introgressed in 1199 suggests that this is the only *B. villosa* allele that is required for the expression of enhanced concentrations of glucoraphanin. The explanation of the high-glucoraphanin trait being determined by a *B. villosa Myb28* allele is entirely consistent with the metabolic analyses which demonstrated that enhanced concentrations of glucoraphanin were due to increased sulphate assimilation and its channelling through to glucoraphanin. Studies in *Arabidopsis* have shown that *Myb28* not only upregulates genes within methionine-derived glucosinolate biosynthesis but also other genes associated with sulphate assimilation and the synthesis of cysteine and methionine (Sonderby *et al*., 2007). Overexpression of Myb28 in *Arabidopsis* also results in altered sulphur partitioning, as the increased concentrations of Met-derived glucosinolates are accompanied by reduced concentrations of glutathione (Yatusevich *et al*., 2010). In *Brassica napus* spp. *oleifera* (canola), it appears that novel *Myb28* alleles for low glucosinolate content (or a *Myb28* deletion) have been introduced into oilseed rape (*B. napus* ssp *oleifera*) to result in lower quantities of 2-hydroxy-3-butenyl glucosinolate (progoitrin) (Harper *et al*., 2012), whereas we have introgressed a *B. villosa* allele of *Myb28* into broccoli to result in enhanced concentrations of 4-methylsulphinylbutyl glucosinolate (glucoraphanin), the precursor of the putative anti-cancer compound sulforaphane.

Glucosinolate concentrations in broccoli are reported to fluctuate with environmental and soil conditions (Bjorkman *et al*., 2011). To demonstrate reproducibility of the high-glucoraphanin phenotype and to understand the impact on yield and quality, extensive experimental field trials were carried out to monitor and validate glucosinolate concentrations in the high-glucoraphanin hybrids in 54 experimental field trials undertaken over 3 years at multiple locations in Arizona, California, Mexico, Spain, Italy and UK. The high-glucoraphanin hybrids including the Beneforté broccoli hybrids were shown to consistently produce 2–3 times the glucoraphanin compared to other leading commercial broccoli varieties while maintaining normal nutrient concentrations (Tables 3, 4), yield and crown quality.

Wild *Brassica* species have previously been used to enhance disease resistance within oilseed rape (Crouch *et al*., 1994; Bradburne *et al*., 1999). In this paper, we describe the use of a wild *Brassica* species to enhance a trait within broccoli with potential consumer benefit. Through the introgression of a *B. villosa Myb28* allele that enhanced sulphate assimilation and specifically channelled the additional sulphur to methionine-derived glucosinolates, we developed commercially viable broccoli F_1_ hybrids with increased concentrations of glucoraphanin, the precursor of sulforaphane. These hybrids are suitable for blinded human intervention studies to investigate the effects of glucoraphanin on human health in a common broccoli matrix without compromising eating quality or other nutritional elements.

## Supporting Information

**Table S1** Content of glucoraphanin in florets of broccoli of 1199, Ironman, Steel and Parthenon in experimental field trials

**Table S2** Content of glucoraphanin in florets of broccoli of 1639, Heritage and Marathon in experimental field trials
