# Supplementary material for: Genetic regulation of glucoraphanin accumulation in Beneforté® broccoli
Source: New Phytol. 2013 Apr 8;198(4):1085–95. doi: 10.1111/nph.12232 (PMC3666090; doi:10.1111/nph.12232)
Supplement: Supplementary file 1 [file nph0198-1085-SD1.docx]

**Table S1** Content of glucoraphanin in florets of broccoli (*Brassica oleracea* L. var *italic* Plenck) of 1199, Ironman, Steel and Parthenon in experimental field trials conducted in 2009, 2010 and 2011

| **Year** | **SubRegion** | **Country** | **Location** | **Trial** | **Variety** | **Glucoraphanin**  **(mg 100g^-1^ fresh wt)** |
| --- | --- | --- | --- | --- | --- | --- |
| 2009 | South | Italy | Lesina | 01_Italy_09 | Ironman | 22.3 |
| 2009 | South | Italy | Lesina | 03_Italy_09 | Ironman | 56.5 |
| 2009 | South | Italy | Lesina | 03_Italy_09 | Ironman | 52 |
| 2009 | South | Italy | Lesina | 03_Italy_09 | Ironman | 59.2 |
| 2009 | South | Italy | Lesina | 03_Italy_09 | Ironman | 63.5 |
| 2009 | South | Spain | Murcia | 02_Spain_09 | Ironman | 17.5 |
| 2009 | South | Spain | Murcia | 04_Spain_09 | Ironman | 33.3 |
| 2009 | South | Spain | Murcia | 04_Spain_09 | Ironman | 53.2 |
| 2009 | South | Spain | Murcia | 04_Spain_09 | Ironman | 33.7 |
| 2009 | South | Italy | Parma | 09_Italy_09 | Ironman | 31 |
| 2009 | South | Italy | Parma | 09_Italy_09 | Ironman | 34.1 |
| 2009 | North | UK | Sibsey | 05_UK_09 | Ironman | 55.2 |
| 2009 | North | UK | Sibsey | 05_UK_09 | Ironman | 68.6 |
| 2009 | North | UK | Sibsey | 06_UK_09 | Ironman | 45.5 |
| 2009 | North | UK | Sibsey | 06_UK_09 | Ironman | 84.2 |
| 2009 | North | UK | Sibsey | 07_UK_09 | Ironman | 41.7 |
| 2009 | North | UK | Sibsey | 07_UK_09 | Ironman | 39.2 |
| 2009 | North | UK | Sibsey | 08_UK_09 | Ironman | 42.2 |
| 2009 | North | UK | Sibsey | 08_UK_09 | Ironman | 44.9 |
|  |  |  |  |  | **Mean ± SD** | **46.2 ± 16.4** |
| 2009 | South | Italy | Lesina | 01_Italy_09 | Parthenon | 11 |
| 2009 | South | Italy | Lesina | 01_Italy_09 | Parthenon | 41.7 |
| 2009 | South | Italy | Lesina | 03_Italy_09 | Parthenon | 49.7 |
| 2009 | South | Italy | Lesina | 03_Italy_09 | Parthenon | 69.8 |
| 2009 | South | Italy | Lesina | 03_Italy_09 | Parthenon | 68.6 |
| 2009 | South | Italy | Lesina | 03_Italy_09 | Parthenon | 56.7 |
| 2009 | South | Spain | Murcia | 02_Spain_09 | Parthenon | 22.7 |
| 2009 | South | Spain | Murcia | 04_Spain_09 | Parthenon | 64.1 |
| 2009 | South | Spain | Murcia | 04_Spain_09 | Parthenon | 56.8 |
| 2009 | South | Spain | Murcia | 04_Spain_09 | Parthenon | 70.5 |
| 2009 | South | Italy | Parma | 09_Italy_09 | Parthenon | 58.1 |
| 2009 | South | Italy | Parma | 09_Italy_09 | Parthenon | 47.6 |
| 2009 | North | UK | Sibsey | 05_UK_09 | Parthenon | 57.3 |
| 2009 | North | UK | Sibsey | 05_UK_09 | Parthenon | 65.9 |
| 2009 | North | UK | Sibsey | 06_UK_09 | Parthenon | 56.9 |
| 2009 | North | UK | Sibsey | 06_UK_09 | Parthenon | 43.5 |
| 2009 | North | UK | Sibsey | 07_UK_09 | Parthenon | 28 |
| 2009 | North | UK | Sibsey | 07_UK_09 | Parthenon | 20.9 |
| 2009 | North | UK | Sibsey | 08_UK_09 | Parthenon | 41.7 |
| 2009 | North | UK | Sibsey | 08_UK_09 | Parthenon | 39 |
|  |  |  |  |  | **Mean ± SD** | **48.5 ± 17.4** |
| 2009 | South | Italy | Lesina | 01_Italy_09 | Steel | 63.1 |
| 2009 | South | Italy | Lesina | 01_Italy_09 | Steel | 53.6 |
| 2009 | South | Italy | Lesina | 03_Italy_09 | Steel | 51.6 |
| 2009 | South | Italy | Lesina | 03_Italy_09 | Steel | 69 |
| 2009 | South | Italy | Lesina | 03_Italy_09 | Steel | 40.3 |
| 2009 | South | Italy | Lesina | 03_Italy_09 | Steel | 51.5 |
| 2009 | South | Spain | Murcia | 02_Spain_09 | Steel | 33 |
| 2009 | South | Spain | Murcia | 04_Spain_09 | Steel | 51.2 |
| 2009 | South | Spain | Murcia | 04_Spain_09 | Steel | 64.3 |
| 2009 | South | Spain | Murcia | 04_Spain_09 | Steel | 58.6 |
| 2009 | South | Italy | Parma | 09_Italy_09 | Steel | 37.1 |
| 2009 | South | Italy | Parma | 09_Italy_09 | Steel | 37.1 |
| 2009 | North | UK | Sibsey | 06_UK_09 | Steel | 69.5 |
| 2009 | North | UK | Sibsey | 06_UK_09 | Steel | 46.7 |
| 2009 | North | UK | Sibsey | 07_UK_09 | Steel | 36.3 |
| 2009 | North | UK | Sibsey | 07_UK_09 | Steel | 32.9 |
| 2009 | North | UK | Sibsey | 08_UK_09 | Steel | 42 |
| 2009 | North | UK | Sibsey | 08_UK_09 | Steel | 39.1 |
|  |  |  |  |  | **Mean ± SD** | **48.7 ± 12.3** |
| 2009 | South | Italy | Lesina | 01_Italy_09 | SVR1199 | 114.1 |
| 2009 | South | Italy | Lesina | 01_Italy_09 | SVR1199 | 45.4 |
| 2009 | South | Italy | Lesina | 03_Italy_09 | SVR1199 | 119.6 |
| 2009 | South | Italy | Lesina | 03_Italy_09 | SVR1199 | 132.3 |
| 2009 | South | Italy | Lesina | 03_Italy_09 | SVR1199 | 153.8 |
| 2009 | South | Italy | Lesina | 03_Italy_09 | SVR1199 | 131.9 |
| 2009 | South | Spain | Murcia | 02_Spain_09 | SVR1199 | 77.4 |
| 2009 | South | Spain | Murcia | 04_Spain_09 | SVR1199 | 123.5 |
| 2009 | South | Spain | Murcia | 04_Spain_09 | SVR1199 | 121.2 |
| 2009 | South | Spain | Murcia | 04_Spain_09 | SVR1199 | 124.8 |
| 2009 | South | Italy | Parma | 09_Italy_09 | SVR1199 | 156 |
| 2009 | South | Italy | Parma | 09_Italy_09 | SVR1199 | 157.8 |
| 2009 | North | UK | Sibsey | 05_UK_09 | SVR1199 | 116.7 |
| 2009 | North | UK | Sibsey | 05_UK_09 | SVR1199 | 136.6 |
| 2009 | North | UK | Sibsey | 06_UK_09 | SVR1199 | 76.5 |
| 2009 | North | UK | Sibsey | 06_UK_09 | SVR1199 | 104.8 |
| 2009 | North | UK | Sibsey | 07_UK_09 | SVR1199 | 89 |
| 2009 | North | UK | Sibsey | 07_UK_09 | SVR1199 | 104.4 |
| 2009 | North | UK | Sibsey | 08_UK_09 | SVR1199 | 118.3 |
| 2009 | North | UK | Sibsey | 08_UK_09 | SVR1199 | 117.8 |
|  |  |  |  |  | **Mean ± SD** | **116.1 ± 28.0** |
| 2010 | South | Spain | Lorqui | 11_Spain_10 | Ironman | 31.5 |
| 2010 | South | Spain | Lorqui | 11_Spain_10 | Ironman | 59.9 |
| 2010 | South | Spain | Lorqui | 12_Spain_10 | Ironman | 46.8 |
| 2010 | South | Spain | Lorqui | 12_Spain_10 | Ironman | 48.9 |
| 2010 | South | Spain | Lorqui | 13_Spain_10 | Ironman | 32.1 |
| 2010 | South | Spain | Lorqui | 13_Spain_10 | Ironman | 29.7 |
| 2010 | South | Spain | Lorqui | 16_Spain_10 | Ironman | 32.7 |
| 2010 | South | Spain | Lorqui | 16_Spain_10 | Ironman | 33.8 |
| 2010 | South | Spain | Lorqui | 17_Spain_10 | Ironman | 83.5 |
| 2010 | South | Spain | Lorqui | 17_Spain_10 | Ironman | 80 |
| 2010 | South | Spain | Murcia | 15_Spain_10 | Ironman | 28.6 |
| 2010 | South | Spain | Murcia | 15_Spain_10 | Ironman | 21.9 |
| 2010 | South | Italy | Parma | 10_Italy_10 | Ironman | 24.9 |
| 2010 | South | Italy | Parma | 10_Italy_10 | Ironman | 29.7 |
| 2010 | South | Italy | Parma | 14_Italy_10 | Ironman | 46.7 |
| 2010 | South | Italy | Parma | 14_Italy_10 | Ironman | 38.1 |
| 2010 | North | UK | Sibsey | 18_UK_10 | Ironman | 32.3 |
| 2010 | North | UK | Sibsey | 18_UK_10 | Ironman | 31.9 |
| 2010 | North | UK | Sibsey | 19_UK_10 | Ironman | 44.1 |
| 2010 | North | UK | Sibsey | 19_UK_10 | Ironman | 45 |
| 2010 | North | UK | Sibsey | 20_UK_10 | Ironman | 64.2 |
| 2010 | North | UK | Sibsey | 20_UK_10 | Ironman | 35.8 |
| 2010 | North | UK | Sibsey | 21_UK_10 | Ironman | 73 |
| 2010 | North | UK | Sibsey | 22_UK_10 | Ironman | 44.4 |
| 2010 | North | UK | Sibsey | 22_UK_10 | Ironman | 49.1 |
| 2010 | North | UK | Sibsey | 23_UK_10 | Ironman | 33.6 |
| 2010 | North | UK | Sibsey | 23_UK_10 | Ironman | 37.6 |
| 2010 | North | UK | Sibsey | 24_UK_10 | Ironman | 32.8 |
| 2010 | North | UK | Sibsey | 24_UK_10 | Ironman | 27.5 |
|  |  |  |  |  | **Mean ± SD** | **42.1 ± 16.1** |
| 2010 | South | Spain | Lorqui | 11_Spain_10 | Parthenon | 45 |
| 2010 | South | Spain | Lorqui | 11_Spain_10 | Parthenon | 41.5 |
| 2010 | South | Spain | Lorqui | 12_Spain_10 | Parthenon | 71.2 |
| 2010 | South | Spain | Lorqui | 12_Spain_10 | Parthenon | 65.1 |
| 2010 | South | Spain | Lorqui | 13_Spain_10 | Parthenon | 45.7 |
| 2010 | South | Spain | Lorqui | 13_Spain_10 | Parthenon | 50.3 |
| 2010 | South | Spain | Lorqui | 16_Spain_10 | Parthenon | 50.4 |
| 2010 | South | Spain | Lorqui | 16_Spain_10 | Parthenon | 38.2 |
| 2010 | South | Spain | Lorqui | 17_Spain_10 | Parthenon | 73.4 |
| 2010 | South | Spain | Lorqui | 17_Spain_10 | Parthenon | 71.2 |
| 2010 | South | Spain | Murcia | 15_Spain_10 | Parthenon | 46.2 |
| 2010 | South | Spain | Murcia | 15_Spain_10 | Parthenon | 47.4 |
| 2010 | South | Italy | Parma | 10_Italy_10 | Parthenon | 38.5 |
| 2010 | South | Italy | Parma | 10_Italy_10 | Parthenon | 36.3 |
| 2010 | South | Italy | Parma | 14_Italy_10 | Parthenon | 42.1 |
| 2010 | South | Italy | Parma | 14_Italy_10 | Parthenon | 46.8 |
| 2010 | North | UK | Sibsey | 18_UK_10 | Parthenon | 45.9 |
| 2010 | North | UK | Sibsey | 18_UK_10 | Parthenon | 45.4 |
| 2010 | North | UK | Sibsey | 19_UK_10 | Parthenon | 50.7 |
| 2010 | North | UK | Sibsey | 19_UK_10 | Parthenon | 45.9 |
| 2010 | North | UK | Sibsey | 20_UK_10 | Parthenon | 37.6 |
| 2010 | North | UK | Sibsey | 21_UK_10 | Parthenon | 52.4 |
| 2010 | North | UK | Sibsey | 22_UK_10 | Parthenon | 43.5 |
| 2010 | North | UK | Sibsey | 22_UK_10 | Parthenon | 38.8 |
| 2010 | North | UK | Sibsey | 23_UK_10 | Parthenon | 31.9 |
| 2010 | North | UK | Sibsey | 23_UK_10 | Parthenon | 32.8 |
| 2010 | North | UK | Sibsey | 24_UK_10 | Parthenon | 28.4 |
| 2010 | North | UK | Sibsey | 24_UK_10 | Parthenon | 31.8 |
|  |  |  |  |  | **Mean ± SD** | **46.2 ± 11.8** |
| 2010 | South | Spain | Lorqui | 11_Spain_10 | Steel | 34.1 |
| 2010 | South | Spain | Lorqui | 11_Spain_10 | Steel | 38.5 |
| 2010 | South | Spain | Lorqui | 12_Spain_10 | Steel | 70.8 |
| 2010 | South | Spain | Lorqui | 12_Spain_10 | Steel | 69.5 |
| 2010 | South | Spain | Lorqui | 13_Spain_10 | Steel | 59 |
| 2010 | South | Spain | Lorqui | 13_Spain_10 | Steel | 56.6 |
| 2010 | South | Spain | Lorqui | 16_Spain_10 | Steel | 38.1 |
| 2010 | South | Spain | Lorqui | 16_Spain_10 | Steel | 36.6 |
| 2010 | South | Spain | Lorqui | 17_Spain_10 | Steel | 62.5 |
| 2010 | South | Spain | Lorqui | 17_Spain_10 | Steel | 69 |
| 2010 | South | Spain | Murcia | 15_Spain_10 | Steel | 53.7 |
| 2010 | South | Spain | Murcia | 15_Spain_10 | Steel | 49.1 |
| 2010 | South | Italy | Parma | 10_Italy_10 | Steel | 39.8 |
| 2010 | South | Italy | Parma | 10_Italy_10 | Steel | 38.9 |
| 2010 | South | Italy | Parma | 14_Italy_10 | Steel | 48.7 |
| 2010 | South | Italy | Parma | 14_Italy_10 | Steel | 51.6 |
| 2010 | North | UK | Sibsey | 18_UK_10 | Steel | 68.6 |
| 2010 | North | UK | Sibsey | 18_UK_10 | Steel | 65.6 |
| 2010 | North | UK | Sibsey | 19_UK_10 | Steel | 58.1 |
| 2010 | North | UK | Sibsey | 19_UK_10 | Steel | 57.2 |
| 2010 | North | UK | Sibsey | 20_UK_10 | Steel | 55.1 |
| 2010 | North | UK | Sibsey | 21_UK_10 | Steel | 60.7 |
| 2010 | North | UK | Sibsey | 22_UK_10 | Steel | 54.8 |
| 2010 | North | UK | Sibsey | 22_UK_10 | Steel | 63.4 |
| 2010 | North | UK | Sibsey | 23_UK_10 | Steel | 23.2 |
| 2010 | North | UK | Sibsey | 23_UK_10 | Steel | 38 |
| 2010 | North | UK | Sibsey | 24_UK_10 | Steel | 37.6 |
| 2010 | North | UK | Sibsey | 24_UK_10 | Steel | 38.3 |
|  |  |  |  |  | **Mean ± SD** | **51.3 ± 13.0** |
| 2010 | South | Spain | Lorqui | 11_Spain_10 | SVR1199 | 109.7 |
| 2010 | South | Spain | Lorqui | 11_Spain_10 | SVR1199 | 118.4 |
| 2010 | South | Spain | Lorqui | 12_Spain_10 | SVR1199 | 139 |
| 2010 | South | Spain | Lorqui | 12_Spain_10 | SVR1199 | 127.2 |
| 2010 | South | Spain | Lorqui | 13_Spain_10 | SVR1199 | 109.8 |
| 2010 | South | Spain | Lorqui | 13_Spain_10 | SVR1199 | 105.5 |
| 2010 | South | Spain | Lorqui | 16_Spain_10 | SVR1199 | 109.9 |
| 2010 | South | Spain | Lorqui | 16_Spain_10 | SVR1199 | 104 |
| 2010 | South | Spain | Lorqui | 17_Spain_10 | SVR1199 | 124.5 |
| 2010 | South | Spain | Lorqui | 17_Spain_10 | SVR1199 | 130.7 |
| 2010 | South | Spain | Murcia | 15_Spain_10 | SVR1199 | 96.9 |
| 2010 | South | Spain | Murcia | 15_Spain_10 | SVR1199 | 112.1 |
| 2010 | South | Italy | Parma | 10_Italy_10 | SVR1199 | 146.4 |
| 2010 | South | Italy | Parma | 10_Italy_10 | SVR1199 | 142.5 |
| 2010 | South | Italy | Parma | 14_Italy_10 | SVR1199 | 145.6 |
| 2010 | South | Italy | Parma | 14_Italy_10 | SVR1199 | 140.8 |
| 2010 | North | UK | Sibsey | 18_UK_10 | SVR1199 | 109.7 |
| 2010 | North | UK | Sibsey | 18_UK_10 | SVR1199 | 97.5 |
| 2010 | North | UK | Sibsey | 19_UK_10 | SVR1199 | 112.3 |
| 2010 | North | UK | Sibsey | 19_UK_10 | SVR1199 | 108.4 |
| 2010 | North | UK | Sibsey | 20_UK_10 | SVR1199 | 125 |
| 2010 | North | UK | Sibsey | 20_UK_10 | SVR1199 | 89.6 |
| 2010 | North | UK | Sibsey | 21_UK_10 | SVR1199 | 148.1 |
| 2010 | North | UK | Sibsey | 22_UK_10 | SVR1199 | 112.9 |
| 2010 | North | UK | Sibsey | 22_UK_10 | SVR1199 | 123 |
| 2010 | North | UK | Sibsey | 23_UK_10 | SVR1199 | 94 |
| 2010 | North | UK | Sibsey | 23_UK_10 | SVR1199 | 95.7 |
| 2010 | North | UK | Sibsey | 24_UK_10 | SVR1199 | 94.8 |
| 2010 | North | UK | Sibsey | 24_UK_10 | SVR1199 | 114.2 |
|  |  |  |  |  | **Mean ± SD** | **116.8 ± 17.4** |
| 2011 | South | Italy | Jesi | 28_Italy_11 | Ironman | 40.3 |
| 2011 | South | Italy | Jesi | 28_Italy_11 | Ironman | 36.2 |
| 2011 | South | Italy | Lavello | 26_Italy_11 | Ironman | 47.7 |
| 2011 | South | Italy | Lavello | 26_Italy_11 | Ironman | 54 |
| 2011 | South | Spain | Lorqui | 25_Spain_11 | Ironman | 23.5 |
| 2011 | South | Spain | Lorqui | 25_Spain_11 | Ironman | 25.1 |
| 2011 | South | Spain | Lorqui | 25_Spain_11 | Ironman | 21.5 |
| 2011 | South | Spain | Lorqui | 27_Spain_11 | Ironman | 34.1 |
| 2011 | South | Spain | Lorqui | 27_Spain_11 | Ironman | 28.6 |
| 2011 | South | Spain | Lorqui | 27_Spain_11 | Ironman | 32.5 |
| 2011 | South | Spain | Lorqui | 29_Spain_11 | Ironman | 24.2 |
| 2011 | South | Spain | Lorqui | 29_Spain_11 | Ironman | 27.6 |
| 2011 | South | Spain | Lorqui | 29_Spain_11 | Ironman | 33.6 |
| 2011 | South | Spain | Lorqui | 30_Spain_11 | Ironman | 29 |
| 2011 | South | Spain | Lorqui | 30_Spain_11 | Ironman | 28 |
| 2011 | South | Spain | Lorqui | 30_Spain_11 | Ironman | 28.1 |
| 2011 | South | Spain | Lorqui | 31_Spain_11 | Ironman | 27.3 |
| 2011 | South | Spain | Lorqui | 31_Spain_11 | Ironman | 36.4 |
| 2011 | South | Spain | Lorqui | 31_Spain_11 | Ironman | 21.3 |
|  |  |  |  |  | **Mean ± SD** | **31.5 ± 8.6** |
| 2011 | South | Italy | Jesi | 28_Italy_11 | Parthenon | 64.1 |
| 2011 | South | Italy | Jesi | 28_Italy_11 | Parthenon | 54.2 |
| 2011 | South | Italy | Lavello | 26_Italy_11 | Parthenon | 73.5 |
| 2011 | South | Italy | Lavello | 26_Italy_11 | Parthenon | 72.4 |
| 2011 | South | Spain | Lorqui | 25_Spain_11 | Parthenon | 37.5 |
| 2011 | South | Spain | Lorqui | 25_Spain_11 | Parthenon | 38.1 |
| 2011 | South | Spain | Lorqui | 25_Spain_11 | Parthenon | 36.8 |
| 2011 | South | Spain | Lorqui | 27_Spain_11 | Parthenon | 45 |
| 2011 | South | Spain | Lorqui | 27_Spain_11 | Parthenon | 34 |
| 2011 | South | Spain | Lorqui | 27_Spain_11 | Parthenon | 44.1 |
| 2011 | South | Spain | Lorqui | 29_Spain_11 | Parthenon | 35 |
| 2011 | South | Spain | Lorqui | 29_Spain_11 | Parthenon | 23.9 |
| 2011 | South | Spain | Lorqui | 29_Spain_11 | Parthenon | 38.9 |
| 2011 | South | Spain | Lorqui | 30_Spain_11 | Parthenon | 65.2 |
| 2011 | South | Spain | Lorqui | 30_Spain_11 | Parthenon | 35.7 |
| 2011 | South | Spain | Lorqui | 30_Spain_11 | Parthenon | 49 |
| 2011 | South | Spain | Lorqui | 31_Spain_11 | Parthenon | 52.6 |
| 2011 | South | Spain | Lorqui | 31_Spain_11 | Parthenon | 40.9 |
| 2011 | South | Spain | Lorqui | 31_Spain_11 | Parthenon | 49.3 |
|  |  |  |  |  | **Mean ± SD** | **46.9 ± 13.8** |
| 2011 | South | Italy | Jesi | 28_Italy_11 | Steel | 48.6 |
| 2011 | South | Italy | Jesi | 28_Italy_11 | Steel | 47.7 |
| 2011 | South | Italy | Lavello | 26_Italy_11 | Steel | 52.9 |
| 2011 | South | Italy | Lavello | 26_Italy_11 | Steel | 50.5 |
| 2011 | South | Spain | Lorqui | 25_Spain_11 | Steel | 31.2 |
| 2011 | South | Spain | Lorqui | 25_Spain_11 | Steel | 29.6 |
| 2011 | South | Spain | Lorqui | 25_Spain_11 | Steel | 31.4 |
| 2011 | South | Spain | Lorqui | 27_Spain_11 | Steel | 22.9 |
| 2011 | South | Spain | Lorqui | 27_Spain_11 | Steel | 23 |
| 2011 | South | Spain | Lorqui | 27_Spain_11 | Steel | 24.7 |
| 2011 | South | Spain | Lorqui | 29_Spain_11 | Steel | 49.3 |
| 2011 | South | Spain | Lorqui | 29_Spain_11 | Steel | 41.7 |
| 2011 | South | Spain | Lorqui | 29_Spain_11 | Steel | 32.5 |
| 2011 | South | Spain | Lorqui | 30_Spain_11 | Steel | 26.8 |
| 2011 | South | Spain | Lorqui | 30_Spain_11 | Steel | 43.7 |
| 2011 | South | Spain | Lorqui | 30_Spain_11 | Steel | 35.4 |
| 2011 | South | Spain | Lorqui | 31_Spain_11 | Steel | 30.3 |
| 2011 | South | Spain | Lorqui | 31_Spain_11 | Steel | 31.2 |
| 2011 | South | Spain | Lorqui | 31_Spain_11 | Steel | 28.6 |
|  |  |  |  |  | **Mean ± SD** | **35.9 ± 10.1** |
| 2011 | South | Italy | Jesi | 28_Italy_11 | SVR1199 | 114 |
| 2011 | South | Italy | Jesi | 28_Italy_11 | SVR1199 | 152.8 |
| 2011 | South | Italy | Lavello | 26_Italy_11 | SVR1199 | 172.7 |
| 2011 | South | Italy | Lavello | 26_Italy_11 | SVR1199 | 121.5 |
| 2011 | South | Spain | Lorqui | 25_Spain_11 | SVR1199 | 77 |
| 2011 | South | Spain | Lorqui | 25_Spain_11 | SVR1199 | 82.5 |
| 2011 | South | Spain | Lorqui | 25_Spain_11 | SVR1199 | 76.3 |
| 2011 | South | Spain | Lorqui | 27_Spain_11 | SVR1199 | 96.5 |
| 2011 | South | Spain | Lorqui | 27_Spain_11 | SVR1199 | 75.3 |
| 2011 | South | Spain | Lorqui | 27_Spain_11 | SVR1199 | 68.1 |
| 2011 | South | Spain | Lorqui | 29_Spain_11 | SVR1199 | 147.8 |
| 2011 | South | Spain | Lorqui | 29_Spain_11 | SVR1199 | 116 |
| 2011 | South | Spain | Lorqui | 29_Spain_11 | SVR1199 | 115.3 |
| 2011 | South | Spain | Lorqui | 30_Spain_11 | SVR1199 | 63.5 |
| 2011 | South | Spain | Lorqui | 30_Spain_11 | SVR1199 | 101.2 |
| 2011 | South | Spain | Lorqui | 30_Spain_11 | SVR1199 | 99.8 |
| 2011 | South | Spain | Lorqui | 31_Spain_11 | SVR1199 | 107.9 |
| 2011 | South | Spain | Lorqui | 31_Spain_11 | SVR1199 | 118.3 |
| 2011 | South | Spain | Lorqui | 31_Spain_11 | SVR1199 | 97.5 |
|  |  |  |  |  | **Mean ± SD** | **105.5 ± 29.5** |

**Table S2** Content of glucoraphanin in florets of broccoli (*Brassica oleracea* L. var *italic* Plenck) of 1639, Heritage and Marathon in experimental field trials conducted in 2007, 2008 and 2010

| **Year** | **Location** | **Grower** | **Variety** | **Glucoraphanin**  **(mg 100g^-1^ fresh wt)** |
| --- | --- | --- | --- | --- |
| 2007 | AGRS,CA | US CA Grower 01 | 1639 | 43.2 |
| 2007 | AGRS,CA | US CA Grower 01 | 1639 | 73.56 |
| 2007 | AGRS,CA | US CA Grower 01 | 1639 | 31.08 |
| 2007 | AGRS,CA | US CA Grower 02 | 1639 | 63.24 |
| 2007 | BAJIO,MX | Mexico Grower 01 | 1639 | 48.72 |
| 2007 | SALINAS,CA | US CA Grower 03 | 1639 | 109.68 |
| 2007 | SALINAS,CA | US CA Grower 04 | 1639 | 85.68 |
| 2007 | SANT A MARIA,CA | US CA Grower 05 | 1639 | 58.56 |
| 2007 | SANT A MARIA,CA | US CA Grower 06 | 1639 | 74.04 |
|  |  |  | **Mean ± SD** | **65.3 ± 23.8** |
| 2007 | AGRS,CA | US CA Grower 01 | 2_Heritage | 34.08 |
| 2007 | AGRS,CA | US CA Grower 01 | 2_Heritage | 8.22 |
| 2007 | AGRS,CA | US CA Grower 02 | 2_Heritage | 26.04 |
| 2007 | BAJIO,MX | Mexico Grower 01 | 2_Heritage | 12.84 |
| 2007 | SALINAS,CA | US CA Grower 03 | 2_Heritage | 66 |
| 2007 | SALINAS,CA | US CA Grower 04 | 2_Heritage | 24.84 |
| 2007 | SANT A MARIA,CA | US CA Grower 05 | 2_Heritage | 27.84 |
| 2007 | SANT A MARIA,CA | US CA Grower 06 | 2_Heritage | 35.76 |
|  |  |  | **Mean ± SD** | **29.5 ± 17.6** |
| 2007 | AGRS,CA | US CA Grower 01 | 3_Marathon | 15.12 |
| 2007 | AGRS,CA | US CA Grower 01 | 3_Marathon | 18 |
| 2007 | AGRS,CA | US CA Grower 01 | 3_Marathon | 26.76 |
| 2007 | AGRS,CA | US CA Grower 02 | 3_Marathon | 31.8 |
| 2007 | BAJIO,MX | Mexico Grower 01 | 3_Marathon | 7.22 |
| 2007 | SALINAS,CA | US CA Grower 03 | 3_Marathon | 37.32 |
| 2007 | SALINAS,CA | US CA Grower 04 | 3_Marathon | 11.05 |
| 2007 | SANT A MARIA,CA | US CA Grower 05 | 3_Marathon | 13.32 |
| 2007 | SANT A MARIA,CA | US CA Grower 06 | 3_Marathon | 23.4 |
|  |  |  | **Mean ± SD** | **20.4 ± 10.1** |
| 2008 | BAJIO,MX | Mexico Grower 02 | 1639 | 47.52 |
| 2008 | CAST ROVILLE,CA | US CA Grower 07 | 1639 | 29.4 |
| 2008 | CAST ROVILLE,CA | US CA Grower 07 | 1639 | 29.4 |
| 2008 | CAST ROVILLE,CA | US CA Grower 07 | 1639 | 36.6 |
| 2008 | SALINAS,CA | US CA Grower 08 | 1639 | 27.48 |
| 2008 | SALINAS,CA | US CA Grower 08 | 1639 | 11.04 |
| 2008 | SALINAS,CA | US CA Grower 08 | 1639 | 28.08 |
| 2008 | SANT A MARIA,CA | US CA Grower 09 | 1639 | 40.44 |
| 2008 | SANT A MARIA,CA | US CA Grower 09 | 1639 | 33.6 |
| 2008 | SANT A MARIA,CA | US CA Grower 09 | 1639 | 35.52 |
| 2008 | YUMA,AZ | US AZ Grower 01 | 1639 | 93.36 |
| 2008 | YUMA,AZ | US AZ Grower 01 | 1639 | 67.44 |
| 2008 | YUMA,AZ | US AZ Grower 01 | 1639 | 74.76 |
| 2008 | YUMA,AZ | US AZ Grower 02 | 1639 | 71.64 |
| 2008 | YUMA,AZ | US AZ Grower 02 | 1639 | 64.2 |
| 2008 | YUMA,AZ | US AZ Grower 02 | 1639 | 72.96 |
|  |  |  | **Mean ± SD** | **47.7 ± 23.1** |
| 2008 | BAJIO,MX | Mexico Grower 02 | 2_Heritage | 10.18 |
| 2008 | CAST ROVILLE,CA | US CA Grower 07 | 2_Heritage | 11.57 |
| 2008 | CAST ROVILLE,CA | US CA Grower 07 | 2_Heritage | 12 |
| 2008 | CAST ROVILLE,CA | US CA Grower 07 | 2_Heritage | 14.88 |
| 2008 | SALINAS,CA | US CA Grower 08 | 2_Heritage | 16.08 |
| 2008 | SALINAS,CA | US CA Grower 08 | 2_Heritage | 11.88 |
| 2008 | SALINAS,CA | US CA Grower 08 | 2_Heritage | 8.04 |
| 2008 | SANT A MARIA,CA | US CA Grower 09 | 2_Heritage | 11.86 |
| 2008 | SANT A MARIA,CA | US CA Grower 09 | 2_Heritage | 17.88 |
| 2008 | SANT A MARIA,CA | US CA Grower 09 | 2_Heritage | 13.44 |
| 2008 | YUMA,AZ | US AZ Grower 01 | 2_Heritage | 34.32 |
| 2008 | YUMA,AZ | US AZ Grower 01 | 2_Heritage | 26.52 |
| 2008 | YUMA,AZ | US AZ Grower 01 | 2_Heritage | 41.4 |
| 2008 | YUMA,AZ | US AZ Grower 02 | 2_Heritage | 29.64 |
| 2008 | YUMA,AZ | US AZ Grower 02 | 2_Heritage | 20.04 |
| 2008 | YUMA,AZ | US AZ Grower 02 | 2_Heritage | 28.32 |
|  |  |  | **Mean ± SD** | **19.3 ±19.8** |
| 2008 | BAJIO,MX | Mexico Grower 02 | 3_Marathon | 4.08 |
| 2008 | CAST ROVILLE,CA | US CA Grower 07 | 3_Marathon | 9.6 |
| 2008 | CAST ROVILLE,CA | US CA Grower 07 | 3_Marathon | 11.95 |
| 2008 | SALINAS,CA | US CA Grower 08 | 3_Marathon | 7.76 |
| 2008 | SALINAS,CA | US CA Grower 08 | 3_Marathon | 6.22 |
| 2008 | SALINAS,CA | US CA Grower 08 | 3_Marathon | 6.88 |
| 2008 | SANT A MARIA,CA | US CA Grower 09 | 3_Marathon | 8.94 |
| 2008 | SANT A MARIA,CA | US CA Grower 09 | 3_Marathon | 7.4 |
| 2008 | SANT A MARIA,CA | US CA Grower 09 | 3_Marathon | 7.91 |
| 2008 | YUMA,AZ | US AZ Grower 01 | 3_Marathon | 16.08 |
| 2008 | YUMA,AZ | US AZ Grower 01 | 3_Marathon | 24.12 |
| 2008 | YUMA,AZ | US AZ Grower 01 | 3_Marathon | 19.44 |
| 2008 | YUMA,AZ | US AZ Grower 02 | 3_Marathon | 18.36 |
|  |  |  | **Mean ± SD** | **11.4 ± 6.1** |
| 2010 | Castroville, CA | US CA Grower 17 | 1639 | 89.71 |
| 2010 | Castroville, CA | US CA Grower 17 | 1639 | 90.6 |
| 2010 | Castroville, CA | US CA Grower 15 | 1639 | 98.71 |
| 2010 | Castroville, CA | US CA Grower 15 | 1639 | 102.66 |
| 2010 | Castroville, CA | US CA Grower 15 | 1639 | 89.52 |
| 2010 | Castroville, CA | US CA Grower 15 | 1639 | 101.1 |
| 2010 | Castroville, CA | US CA Grower 15 | 1639 | 121.92 |
| 2010 | Castroville, CA | US CA Grower 15 | 1639 | 106.5 |
| 2010 | Chualar | US CA Grower 14 | 1639 | 66.03 |
| 2010 | Chualar | US CA Grower 14 | 1639 | 81.35 |
| 2010 | Chualar | US CA Grower 14 | 1639 | 101.05 |
| 2010 | Chualar | US CA Grower 14 | 1639 | 72.47 |
| 2010 | Chualar | US CA Grower 14 | 1639 | 89.13 |
| 2010 | Chualar | US CA Grower 14 | 1639 | 94.76 |
| 2010 | north of Salinas | US CA Grower 12 | 1639 | 77.49 |
| 2010 | north of Salinas | US CA Grower 12 | 1639 | 82.09 |
| 2010 | north of Salinas | US CA Grower 12 | 1639 | 59.03 |
| 2010 | north of Salinas | US CA Grower 12 | 1639 | 73.04 |
| 2010 | north of Salinas | US CA Grower 12 | 1639 | 56.72 |
| 2010 | north of Salinas | US CA Grower 12 | 1639 | 70.26 |
| 2010 | north of Salinas | US CA Grower 11 | 1639 | 81.34 |
| 2010 | north of Salinas | US CA Grower 11 | 1639 | 82.2 |
| 2010 | north of Salinas | US CA Grower 11 | 1639 | 96.1 |
| 2010 | north of Salinas | US CA Grower 11 | 1639 | 92.7 |
| 2010 | north of Salinas | US CA Grower 11 | 1639 | 74.63 |
| 2010 | north of Salinas | US CA Grower 11 | 1639 | 92.88 |
| 2010 | Pajaro, CA | US CA Grower 16 | 1639 | 71.46 |
| 2010 | Pajaro, CA | US CA Grower 16 | 1639 | 64.44 |
| 2010 | Pajaro, CA | US CA Grower 16 | 1639 | 58.46 |
| 2010 | Pajaro, CA | US CA Grower 16 | 1639 | 68.38 |
| 2010 | Pajaro, CA | US CA Grower 16 | 1639 | 72.04 |
| 2010 | Pajaro, CA | US CA Grower 16 | 1639 | 63.22 |
| 2010 | Pajaro, CA | US CA Grower 10 | 1639 | 169.62 |
| 2010 | Pajaro, CA | US CA Grower 10 | 1639 | 158.99 |
| 2010 | Pajaro, CA | US CA Grower 10 | 1639 | 145.12 |
| 2010 | Pajaro, CA | US CA Grower 10 | 1639 | 147.51 |
| 2010 | Pajaro, CA | US CA Grower 10 | 1639 | 132.16 |
| 2010 | Pajaro, CA | US CA Grower 10 | 1639 | 224.77 |
| 2010 | San Ysidro Farms, Nip | oUS CA Grower 18 | 1639 | 52.06 |
| 2010 | San Ysidro Farms, Nip | US CA Grower 18 | 1639 | 130.66 |
| 2010 | San Ysidro Farms, Nip | oUS CA Grower 18 | 1639 | 139.83 |
| 2010 | San Ysidro Farms, Nip | oUS CA Grower 19 | 1639 | 83.88 |
| 2010 | south of Salinas | US CA Grower 13 | 1639 | 77.77 |
| 2010 | south of Salinas | US CA Grower 13 | 1639 | 75.29 |
| 2010 | south of Salinas | US CA Grower 13 | 1639 | 65.09 |
| 2010 | south of Salinas | US CA Grower 13 | 1639 | 63.9 |
|  |  |  | **Mean ± SD** | **93.7 ± 34.5** |
| 2010 | Castroville, CA | US CA Grower 17 | 2_Heritage | 22.14 |
| 2010 | Castroville, CA | US CA Grower 17 | 2_Heritage | 21.93 |
| 2010 | Castroville, CA | US CA Grower 15 | 2_Heritage | 47.55 |
| 2010 | Castroville, CA | US CA Grower 15 | 2_Heritage | 32.15 |
| 2010 | Castroville, CA | US CA Grower 15 | 2_Heritage | 35.96 |
| 2010 | Castroville, CA | US CA Grower 15 | 2_Heritage | 36.55 |
| 2010 | Castroville, CA | US CA Grower 15 | 2_Heritage | 50.67 |
| 2010 | Castroville, CA | US CA Grower 15 | 2_Heritage | 32.84 |
| 2010 | Chualar | US CA Grower 14 | 2_Heritage | 34.07 |
| 2010 | Chualar | US CA Grower 14 | 2_Heritage | 25.8 |
| 2010 | Chualar | US CA Grower 14 | 2_Heritage | 24.45 |
| 2010 | Chualar | US CA Grower 14 | 2_Heritage | 28.5 |
| 2010 | Chualar | US CA Grower 14 | 2_Heritage | 31.5 |
| 2010 | Chualar | US CA Grower 14 | 2_Heritage | 31.65 |
| 2010 | north of Salinas | US CA Grower 12 | 2_Heritage | 29.62 |
| 2010 | north of Salinas | US CA Grower 12 | 2_Heritage | 38.18 |
| 2010 | north of Salinas | US CA Grower 12 | 2_Heritage | 32.22 |
| 2010 | north of Salinas | US CA Grower 12 | 2_Heritage | 38.92 |
| 2010 | north of Salinas | US CA Grower 11 | 2_Heritage | 37.12 |
| 2010 | north of Salinas | US CA Grower 11 | 2_Heritage | 36.42 |
| 2010 | north of Salinas | US CA Grower 11 | 2_Heritage | 39.34 |
| 2010 | north of Salinas | US CA Grower 11 | 2_Heritage | 29.17 |
| 2010 | north of Salinas | US CA Grower 11 | 2_Heritage | 36.11 |
| 2010 | north of Salinas | US CA Grower 11 | 2_Heritage | 50.22 |
| 2010 | Pajaro, CA | US CA Grower 16 | 2_Heritage | 26.41 |
| 2010 | Pajaro, CA | US CA Grower 16 | 2_Heritage | 28.71 |
| 2010 | Pajaro, CA | US CA Grower 16 | 2_Heritage | 27.86 |
| 2010 | Pajaro, CA | US CA Grower 16 | 2_Heritage | 15.17 |
| 2010 | Pajaro, CA | US CA Grower 16 | 2_Heritage | 20.25 |
| 2010 | Pajaro, CA | US CA Grower 16 | 2_Heritage | 23.19 |
| 2010 | Pajaro, CA | US CA Grower 10 | 2_Heritage | 66.45 |
| 2010 | Pajaro, CA | US CA Grower 10 | 2_Heritage | 80.77 |
| 2010 | Pajaro, CA | US CA Grower 10 | 2_Heritage | 73.5 |
| 2010 | Pajaro, CA | US CA Grower 10 | 2_Heritage | 59.95 |
| 2010 | San Ysidro Farms, Nip | US CA Grower 18 | 2_Heritage | 55.86 |
| 2010 | San Ysidro Farms, Nip | oUS CA Grower 18 | 2_Heritage | 39.64 |
| 2010 | San Ysidro Farms, Nip | oUS CA Grower 18 | 2_Heritage | 58.15 |
| 2010 | San Ysidro Farms, Nip | US CA Grower 19 | 2_Heritage | 25.64 |
| 2010 | south of Salinas | US CA Grower 13 | 2_Heritage | 20.46 |
| 2010 | south of Salinas | US CA Grower 13 | 2_Heritage | 24.12 |
| 2010 | south of Salinas | US CA Grower 13 | 2_Heritage | 20.91 |
| 2010 | south of Salinas | US CA Grower 13 | 2_Heritage | 36.23 |
|  |  |  | **Mean ± SD** | **36.3 ± 14.8** |
| 2010 | San Ysidro Farms, Nip | US CA Grower 18 | 3_Marathon | 12.12 |
| 2010 | San Ysidro Farms, Nip | oUS CA Grower 18 | 3_Marathon | 13.43 |
| 2010 | San Ysidro Farms, Nip | US CA Grower 18 | 3_Marathon | 25.84 |
|  |  |  | **Mean ± SD** | **17.1 ± 7.6** |
